# Integrated miRNA and mRNA expression profiling in fetal hippocampus with Down syndrome

**DOI:** 10.1186/s12929-016-0265-0

**Published:** 2016-06-07

**Authors:** Wei-li Shi, Zhong-zhen Liu, Hong-dan Wang, Dong Wu, Hui Zhang, Hai Xiao, Yan Chu, Qiao-fang Hou, Shi-xiu Liao

**Affiliations:** Medical Genetic Institute of Henan Province, Henan Provincial People’s Hospital, People’s Hospital of Zhengzhou University, Zhengzhou, People’s Republic of China; Key Laboratory for Regenerative Medicine, Ministry of Education, School of Biomedical Sciences, Faculty of Medicine, The Chinese University of Hong Kong, Hong Kong SAR, People’s Republic of China

**Keywords:** Down syndrome (DS), Hippocampus, Intellectual disability

## Abstract

**Backgrounds:**

Down syndrome (DS), caused by triplication of human chromosome 21, is the most common aneuploidies. The main characteristic of DS patients is intellectual disability. MicroRNAs (miRNAs) play important regulatory roles in various biological processes, such as embryonic development, cell differentiation, proliferation and apoptosis. Several miRNAs have shown association with DS. However, the role of miRNAs in DS patients has not been well elaborated.

**Methods:**

In this research, total RNA extracted from fetal hippocampal tissues was used to analyze miRNA and mRNA expression via Affymetrix miRNA 4.0 and PrimeView Human Gene Expression Array, respectively. Then miRNA and gene expression profiles were integrated by correlation analysis to identify dysregulated miRNAs with their predicted target mRNAs. Microarray data were further validated by real-time PCR. Regulation of zeste homolog 2 (EZH2) expression by hsa-miR-138 was determined by luciferase reporter assay.

**Results:**

We analyzed microRNA expression profile in hippocampal tissues from DS fetal using miRNA microarray. Further with the use of mRNA microarray data, we integrate miRNA expression and mRNA expression in hippocampus of trisomy 21 fetus to elucidate the mechanism that underlying DS abnormalities. We characterized the repertoire of specific miRNAs involved in hippocampus in trisomy 21 patients, highlighting hsa-miR-138 and hsa-miR-409, in particular the importance of hsa-miR-138, especially the -5p strand. Furthermore, the expression level of predicted target genes of hsa-miR-138-5p in trisomy 21 fetus, including zeste homolog 2 (EZH2) were further confirmed. In addition, luciferase assay indicated that EZH2 was a direct target of hsa-miR-138 in HEK293T cells.

**Conclusion:**

The function of hsa-miR-138-5p and its target EZH2 was involved in hippocampus in DS patients. Our findings provide a clue to study the underlying molecular mechanisms in DS patients, and its potential contribution in improving understanding of intellectual disability development in DS patients.

**Electronic supplementary material:**

The online version of this article (doi:10.1186/s12929-016-0265-0) contains supplementary material, which is available to authorized users.

## Background

Down syndrome (DS), or trisomy 21 [MIM: 190685], is a common chromosomal abnormality. This disease is caused by an extra copy of chromosomal 21 and therefore called trisomy. The incidence of DS is about 1:700 births and the incidence increases with high maternal age [1]. There are various phenotypes in DS population, including craniofacial abnormality, learning disabilities, congenital heart disease, leukemia’s, Alzheimer’s disease and a variety of physical features such as slanted eye, abnormal pattern of fingerprint, large tongue [2, 3]. The main neurological deficiency in DS patients is intellectual disability and early onset of Alzheimer’s disease [4]. So far, several genes have been associated with Alzheimer’s disease in DS patients, such as APP [5], BACE2 [6] and PICALM [7]. However, the role of miRNA in intellectual disability of DS patients is largely unknown. Elucidation of neurological deficiency in DS patients will contribute to better understand of this disease.

MicroRNAs (miRNAs) are small single stranded noncoding RNAs. MiRNAs regulate gene expression post-transcriptionally via binding with complementary targets resulting in inhibition of target mRNA translation [8–10]. Increasing evidence indicates that miRNAs play important roles in a large variety of biological processes such as development, differentiation, proliferation and apoptosis [11]. So far, several miRNAs have been implicated in Down syndrome. For example, hsa-miR-155 contributes to reduce the incidence of hypertension in DS patients [12].

Fetal hippocampus consists of Ammon’s horn, dentate gyrus, entorhinal cortex, parasubiculum, presubiculum, and subicular complex. Hippocampus is involved in spatial learning, short-term memory and long-term memory [13]. Opperman et al. have showed the difference protein expression in fetal human brain between DS and control subjects [14]. However, the function of miRNAs in DS is still largely undefined. The study of miRNA on hippocampus of DS patients will shed light on the mechanism that underlying neurological deficiency in DS patients.

In this study, we tried to establish a comprehensive correlation between the miRNA expression and target gene regulation in hippocampus in Down syndrome by integration of miRNA and mRNA expression data. The results implicated that hsa-miR-138 and its targets enhancer of zeste homolog 2 (EZH2) were involved in DS patients and potentially contributed in neurological deficiency in DS patients.

## Methods

### Sample preparation

Four fetal brains that were diagnosed as trisomy 21 by amniocentesis analysis were obtained at abortions in the pregnancy of 16–20 weeks. The other three were obtained at spontaneous abortions in the pregnancy of 16–20 weeks and were diagnosed as diploid controls. The hippocampus in fetal were quickly dissected out from the surrounding tissues, and then were frozen in liquid nitrogen.

### RNA isolation

Total RNA of fetal hippocampal tissues of DS (*n* = 4) and diploid controls (*n* = 3) was extracted respectively using the Trizol reagent (Invitrogen) according to manufacture’s protocol as we published previously [15].

### MiRNA microarray analysis

Total RNA of DS (*n* = 3) and diploid controls (*n* = 3) was purified by mirVana miRNA Isolation Kit (Ambion). The miRNA expression profiles were generated by using the Affymetrix GeneChip miRNA Array v. 4.0 (Affymetrix). Briefly, the flashTag Biotin RNA Labeling Kit (Affymetrix) was used to label of 1 μg of total RNA, followed by the hybridization overnight according to the manufacturer’s instructions. After washed and stained using the Affymetrix GeneChip Hybridization Wash and Stain Kit, the miRNA chips were then scanned with the Affymetrix GeneChip Scanner 3000 (Affymetrix).

### mRNA microarray analysis

Total RNA (0.1 μg) of DS (*n* = 3) and diploid controls (*n* = 3) was employed for the expression analysis with PrimeView Human Gene Expression Array (Affymetrix). RNA was labeled by biotin using the GeneChip 3′ IVT labeling kit (Affymetrix). After hybridization overnight, the chips were washed and stained by GeneChip Hybridization, wash, and stain Kit (Affymetrix) according to the manufacturer’s protocols. The hybridized chips were scanned by GeneChip Scanner 3000.

### Microarray expression data Analysis

The CEL-files of the raw data were obtained by Affymetrix GeneChip Command Console Software (Affymetrix). SAM (significance analysis of microarray) with the R package was used to identify differentially expressed miRNA and gene probe sets between DS fetal hippocampus and controls. Probe sets were considered as biologically significant if the fold changes (FC) are over 2 or less than 0.05 and a false discovery rate (FDR) < 0.05.

### Prediction of target mRNAs of miRNAs

We predicted the target mRNAs of the miRNA using the bioinformatics prediction tool including miRWalk, miRanda, miRDB, RNA, DIANAmT, RNAhybrid, PICTAR4, PICTAR5, PITA, RNA22 and TargetScan. The selection criteria is correlation > 0.99 or correlation < −0.99, and p value < 0.05.

### Detection of miRNA expression with real-time PCR

Total RNA of DS (*n* = 4) and diploid controls (*n* = 3) was extracted respectively by Trizol reagent, followed by purification by mirVana miRNA Isolation Kit, and then was reversely transcribed into cDNA by M-MLV Reverse Transcriptase (Invitrogen). Real-time PCR was carried out using SYBR Green master mix (Applied Biosystems) according to the manufacturer’s instructions on a 7500 Fast Real-Time PCR system (Applied Biosystems) and analyzed with the SDS analysis software package (version 2.0.1, Applied Biosystems). Samples were analyzed in triplicate and all the PCR primers were purchased from Invitrogen (Table 1). The U6 snRNA was used as the internal standard control.

**Table 1:** Primer pairs used for quantitative real-time PCR

Name	Sequence (5′- 3′)
MiRNA Universal Primer	GTGCAGGGTCCGAGGT
hsa-miR-138-5p-RT	GTCGTATCCAGTGCAGGGTCCGAGG
	TATTCGCACTGGATACGACcggcct
hsa-miR-138-5p-AS	GCATAGCTGGTGTTGTGAATCAG
hsa-miR-409-5p-RT	GTCGTATCCAGTGCAGGGTCCGAGG
	TATTCGCACTGGATACGACatgcaa
hsa-miR-409-5p-AS	TCAGGTTACCCGAGCAACTTT
hsa-miR-19-3p-RT	GTCGTATCCAGTGCAGGGTCCGAGG
	TATTCGCACTGGATACGACtcagtt
hsa-miR-19-3p-AS	GCTGTGCAAATCCATTCAA
hsa-miR-204-5p-RT	GTCGTATCCAGTGCAGGGTCCGAGG
	TATTCGCACTGGATACGACaggcat
hsa-miR-204-5p-AS	GCTTCCCTTTGTCATCCT
U6 Fw	CTCGCTTCGGCAGCACA
U6 Re	AACGCTTCACGAATTTGCGT
MAML1 Fw	TCCTCACTTGCTGCAGTGTC
MAML1 Re	CTCCCCTGACTCCCGAAAAC
NNAT Fw	GGCCGTATCATCAGGTGCTC
NNAT Re	TCTACTGGCCCCTCACTGAC
SOX11 Fw	TAGCCACTCCAGGTTTGGGA
SOX11 Re	CTGTTTCACAGCAATAGGCCG
EZH2 Fw	CCATCCAGACTGGCGAAGAG
EZH2 Re	AGGCAGCTGTTTCAGAGGAG
VEZF1 Fw	GCCTCAACTGACAGAGGAGAAG
VEZF1 Re	TCCAAAGTACTCCTTGGCTCC
ITGB3BP Fw	ACCAACTTCTGCATCTGCCT
ITGB3BP Re	TAGACATGCACCTGCCAACT
CBX2 Fw	CAAGTGCCGTCACTGGGAA
CBX2 Re	CGTCAGAAGTGCCAGATACCTA
EFNB1 Fw	TGTGCACTTTGACCCCAGTT
EFNB1 Re	GTGGAGGTGGATGGTGTGAG
SMARCC1 Fw	CTGGAATACGGGAACCGAGG
SMARCC1 Re	TTGTCAAAAGGCACTGGGGT
PCP4 Fw	ATGAGTGAGCGACAAGGTG
PCP4 Re	TTCAGGTGGACTAGGAGGG
C2CD2 Fw	GAAGACTCCCATCAAGGTG
C2CD2 Re	GATCCCTGATATGATAATAGTGC
APP Fw	CCGACCGAGGACTGACCACT
APP Re	TGACAACACCGCCCACCAT
MX1 Fw	GGTCAGTTACCAGGACTACGA
MX1 Re	GTTATGCCAGGAAGGTCTATT
PDXK Fw	TTGTGCAGGAGCTGAAGCA
PDXK Re	GGTTGGGCGTGATAATGTCT
AGPAT3 Fw	TCTACGGGAAGAAGTACGAGG
AGPAT3 Re	GACAGGAAGTTCAGGAGGGT
GAPDH Fw	CATGTACGTTGCTATCCAGGC
GAPDH Re	CTCCTTAATGTCACGCACGAT

*RT* Reverse transcript primer, *AS* anti-sense primer, *Fw* forward primer, *Re* reverse primer

### Detection of mRNA expression with real-time PCR

The real-time PCR to detect mRNA expression was carried out as we published previously [15]. Briefly, the total RNA of DS (*n* = 4) and diploid controls (*n* = 3) was respectively transcribed by M-MLV Reverse Transcriptase (Invitrogen), real-time PCR was run in a final reaction volume of 20 μl by SYBR Green master mix (Applied Biosystems) on the Applied Biosystems 7500 Fast Real-Time PCR System. All samples were analyzed in triplicate and all the PCR primers were ordered from Invitrogen (Table 1). GAPDH served as the reference.

### Real-time PCR results analysis

Real-time PCR results were calculated using the 2^-ΔΔCt^ method. The ratio of expression in DS hippocampal tissues relative to the control tissues more than 1.0 was considered as obvious increased and less than 1.0 was considered as decreased.

### Plasmid construction and Luciferase reporter assay

The 3′UTR of EZH2 was amplified by PCR from genomic DNA of HEK293T cells with the following primers: Forward: 5′ AGGTCTAGACCTCTGAAACAGCTGCCTTA 3′ and Reverse: 5′ CCTGAGCTCGCATTATTGCAAAAATTCAC 3′. The 3′UTR was cloned downstream of the luciferase coding sequence in the pMIR-REPORT Luciferase vector (Promega). The construct was confirmed by sequencing. Hsa-miR-138 mimics and none-target control were purchased from GenePharma. And the sequence are as follows: hsa-miR-138 mimics, Forward: 5′AGCUGGUGUUGUGAAUCAGGCCG 3′and Reverse: 5′GCCUGAUUCACAACACCAGCUUU 3′; none-target control, Forward: 5′ UUCUCCGAACGUGUCACGUTT 3′and Reverse: 5′ ACGUGACACGUUCGGAGAATT 3′.

HEK293T cells were plated in 24-well dishes overnight before transfection. Hsa-miR-138 mimics or none-target control was transfected into HEK293T cells as well as 200 ng of pMIR-REPORT-EZH2 and 20 ng of pRL-renilla by lipofectmine 2000 (Invitrogen). Medium was changed 6 h later. After 48 h, luciferase assay was performed with dual-luciferase reporter assay systems (Promega) according to the manufacturer’s instructions. Measurements were carried out in triplicates and expressed as mean ± SD. Three independent transfection experiments were performed.

### Statistics analysis

Statistical analysis was performed using SPSS 13.0 software. The values are presented as mean ± SD. The differences between two groups were calculated by Student’s t-test. *P* < 0.05 was considered as statistically significant difference.

## Results

### Differentially expressed miRNAs in DS group versus control group

**Fig. 1:**
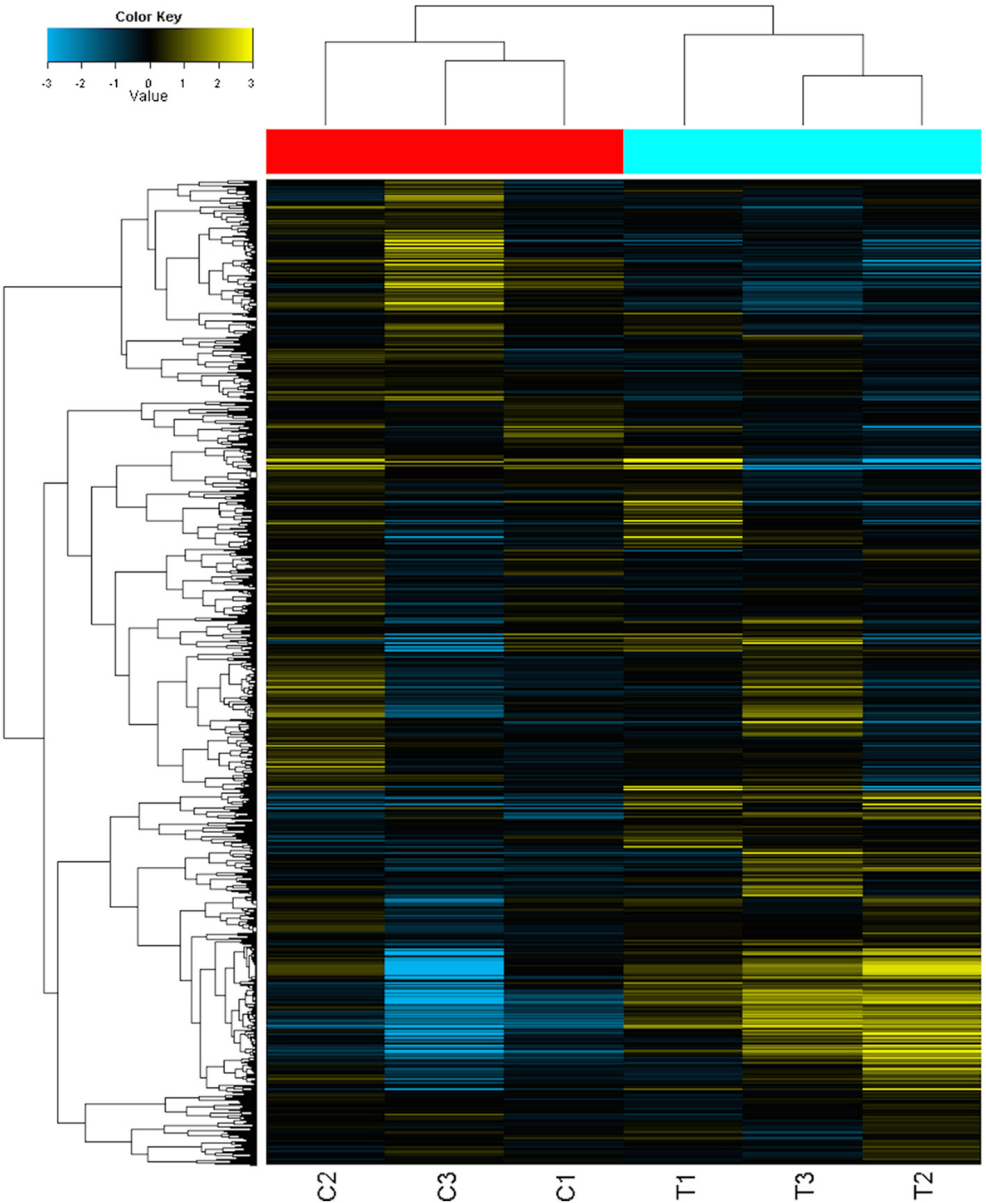
Hierarchical clustering of DS fetal samples and control samples. Hierarchical clustering of all covered human mature miRNAs. Samples are shown in the columns and miRNAs in the rows. The boxes in color indicate the log2 intensities of the miRNAs, with blue indicating low expression and yellow indicating high expression

MiRNA expression profiles were compared between DS group and control group. Of all the miRNAs measured, seventy-two mature miRNAs that were significantly up-regulated with fold changes over than 2, and five down-regulated were identified in DS group versus control group (Additional file 1: Table S1). The hierarchical cluster of all covered mature miRNAs was showed in Fig. 1.

### Differentially expressed mRNAs in DS group versus control group

**Fig. 2:**
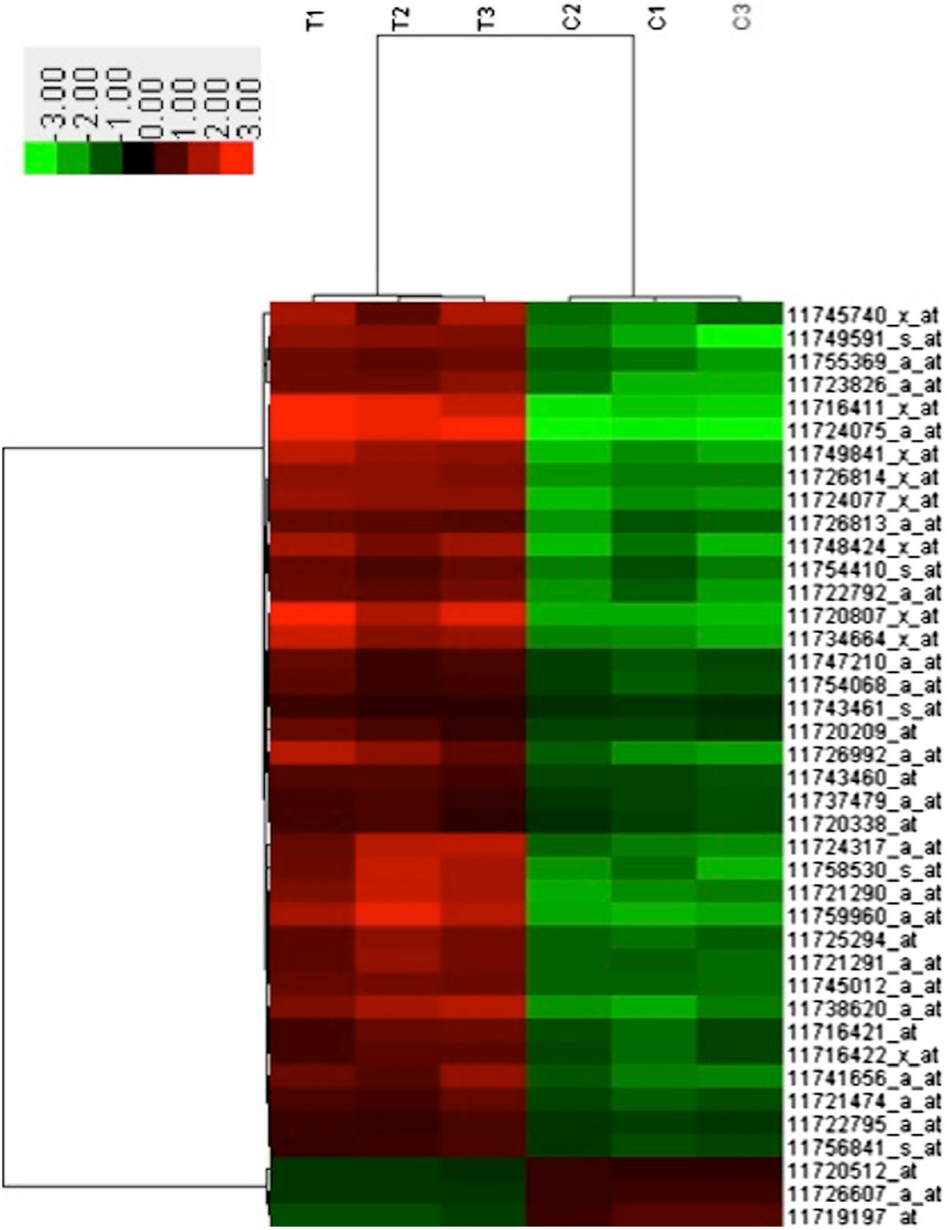
Heatmap of mRNA expression in fetal hippocampus of DS fetal cohorts and control cohorts. Hierarchical clustering of all samples based on the log2 expression values of the top 40 most variable mRNAs. Samples are shown in the columns and mRNAs in the rows. The boxes in color indicate the log2 intensities of the mRNAs, with green indicating low expression and red indicating high expression

In order to identify potential targets of the changed miRNAs, the mRNA expression profile of the same hippocampal tissues also was investigated with Affymetrix microarray. The results showed that 1160 mRNA were significantly increased and 994 mRNA were significantly decreased in DS group. And 51 up-regulated mRNA were from chromosome 21 (Table 2). Cluster analysis of top 40 probe sets encoding mRNAs with the highest variation in expression across the 6 samples showed two major clusters (Fig. 2). And cluster analysis of all the differentially expressed mRNA was shown in Additional file 2: Figure S1.

**Table 2:** The increased mRNA located on chromosome 21

Gene symbol	Fold change	*P* value (%)
MX1	7.3078	0.827529807
PCP4	7.2605	0.899717682
S100B	6.5814	3.469117486
SAMSN1	5.4615	2.683368388
OLIG1	5.408	4.532988776
C2CD2	4.2167	0.146064819
C21orf33	4.0585	0.146064819
SH3BGR	3.5808	1.597151208
APP	3.5603	3.469117486
TMEM50B	3.5265	0.377152444
AGPAT3	3.2927	0.961462767
C21orf88	3.2137	2.088087924
PDXK	3.0927	2.332542808
HLCS	2.8104	1.909735373
BRWD1	2.6731	2.295354625
OLIG2	2.6037	4.632689187
SMIM11	2.5938	0.805146516
WRB	2.5279	1.788040099
NCAM2	2.5126	2.583461784
PTTG1IP	2.5087	0.146064819
PIGP	2.399	0.408886954
SYNJ1	2.3636	0.986344775
ERG	2.2802	4.111356376
CXADR	2.269	3.664851129
CBR1	2.2509	0.986344775
IFNAR1	2.1416	0.794175289
TIAM1	2.1345	0.843185443
SUMO3	2.1021	1.188403617
ADARB1	2.0513	4.931606353
PSMG1	2.0493	2.426143122

The GO-enrichment analysis was used to illustrate the biological functions to the putative target mRNAs. The target genes are predominantly involved in the biological functions including cell cycle, meiosis, gap junction, fructose and mannose metabolism and alanine, aspartate and glutamate metabolism (Table 3).

**Table 3:** 15 pathways significantly influenced by differentially expressed RNAs

KEGG pathway	Genes	*P* value
Cell cycle	428	9.4362E-09
Cell cycle - yeast	278	1.55857E-08
Gap junction	304	4.49918E-08
Oocyte meiosis	472	2.65787E-06
Progesterone-mediated oocyte maturation	346	4.63607E-05
Long-term depression	269	5.33789E-05
Prion diseases	134	0.000130518
Salivary secretion	409	0.000200115
Melanoma	249	0.000274233
Wnt signaling pathway	679	0.000387352
p53 signaling pathway	274	0.000446442
Fructose and mannose metabolism	124	0.001832756
Meiosis - yeast	259	0.002555986
Bacterial invasion of epithelial cells	440	0.002752716
Alanine, aspartate and glutamate metabolism	101	0.002969861

*Genes* number of genes involved in the KEGG pathway

### Correlation between expression levels of significantly differentially expressed miRNAs and their predicted targets mRNAs

**Fig. 3:**
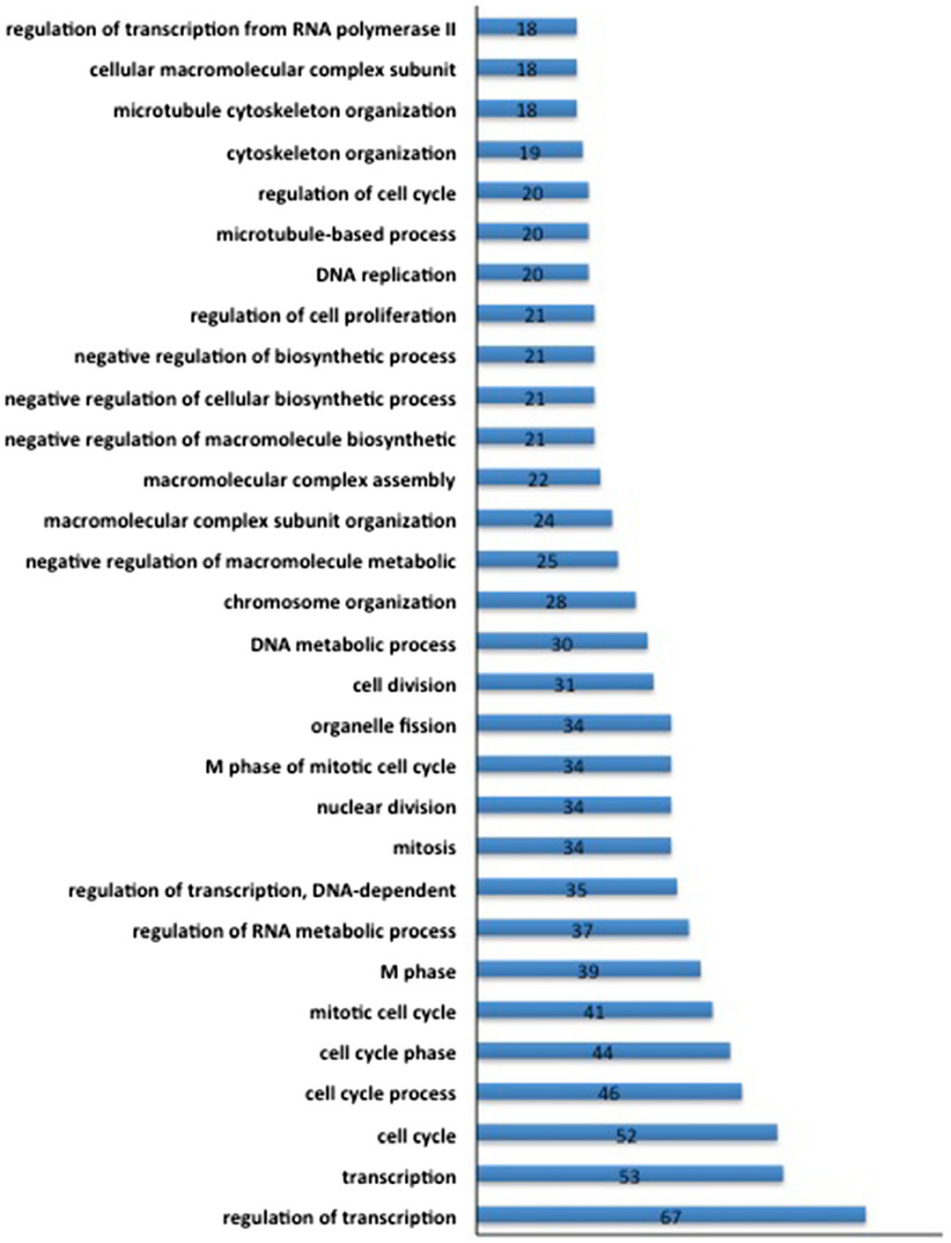
Enrichment of biological processes in term of Gene Ontology categories with predicted miRNA-mRNA pairs. A number of biological processes were enrichment with predicted miRNA-mRNA pairs. The number of genes in each GO category is indicated within the plot

**Fig. 4:**
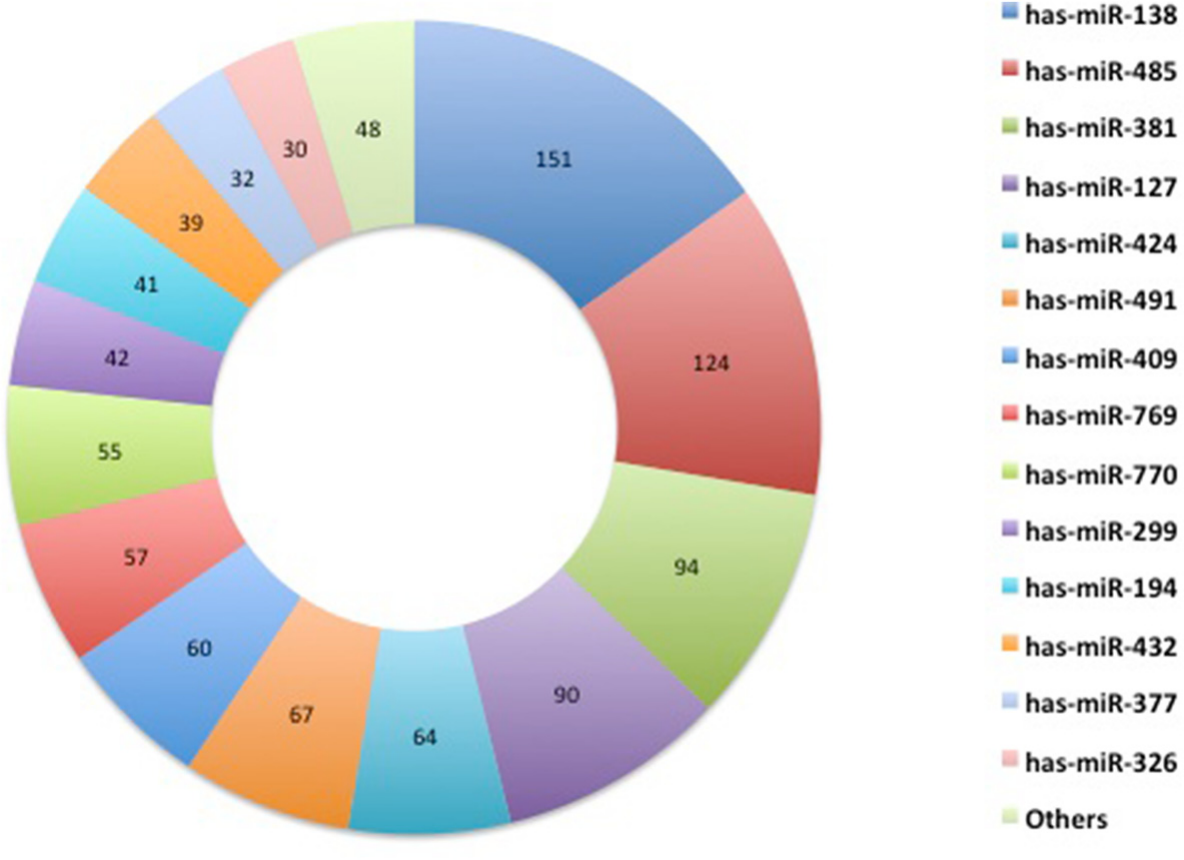
The proportion of miRNAs that involved in predicted miRNA-mRNA pairs. The number of predicted miRNA-mRNA pairs of each miRNA was indicated within the plot. Hsa-miR-138 had the maximum predicted target mRNAs among all the pairs

In order to identify target mRNAs of one or more of the differentially expressed miRNAs that are potentially related with DS fetal neurological deficiency, we applied two criteria, 1) the target mRNAs should be differentially co-expressed together with the altered miRNAs in DS fetal hippocampal tissues; and 2) the target mRNA should display an inverse expression correlation with the miRNA. Total 995 pairs of significantly altered miRNAs and predicted target mRNAs fulfilled the criteria in DS fetal group versus controls. Functional analysis indicated that the gene list was significantly enriched in regulation of transcription (Fig. 3). The proportion of significantly altered miRNAs that involved in predicted miRNA-target mRNAs pairs was shown in Fig. 4. The top 10 miRNA - target mRNA pairs that have the largest number of predicted results in prediction tools were listed in Table 4. Among them, seven of the top 10 miRNA-target mRNA pairs are related to hsa-miR-138. Cytoscape software suggested that hsa-miR-138 has the largest number of mRNA neighbors. This miRNA was chosen to validate microarray results by real time-PCR, along with its predicted target mRNAs: *MAML1, NNAT, VEZF1, ITGB3BP, SOX11, EZH2* and *CBX2*. Of all miRNA-mRNA pairs, we also selected hsa-miR-409-5p, which played roles in cell proliferation and apoptosis [16], along with its predicted target genes, *SMARCC1* and *EFNB1*, and another two miRNAs, hsa-miR-19-3p and hsa-miR-204-5p to validate the microarray results.

**Table 4:** Top 10 most significantly correlated miRNA-mRNA pairs

MiRNAs	Predicated target	Correlation	*P*. value	SUM
hsa-miR-138	EZH2	−1	0.002777778	9
hsa-miR-138	DVL2	−1	0.002777778	7
hsa-miR-485-3p	TRIM24	−1	0.002777778	7
hsa-miR-138	ARRDC3	−1	0.002777778	7
hsa-miR-138	DVL2	−1	0.002777778	7
hsa-miR-138	NNAT	−1	0.002777778	7
hsa-miR-138	VEZF1	−1	0.002777778	7
hsa-miR-770-5p	BTG1	−1	0.002777778	7
hsa-miR-138	NNAT	−1	0.002777778	7
hsa-miR-770-5p	BTG1	−1	0.002777778	7

*SUM* The total of predicated miRNA-mRNA pairs in ten different prediction tools

### Validation of the microarray expression data by real-time PCR

**Fig. 5:**
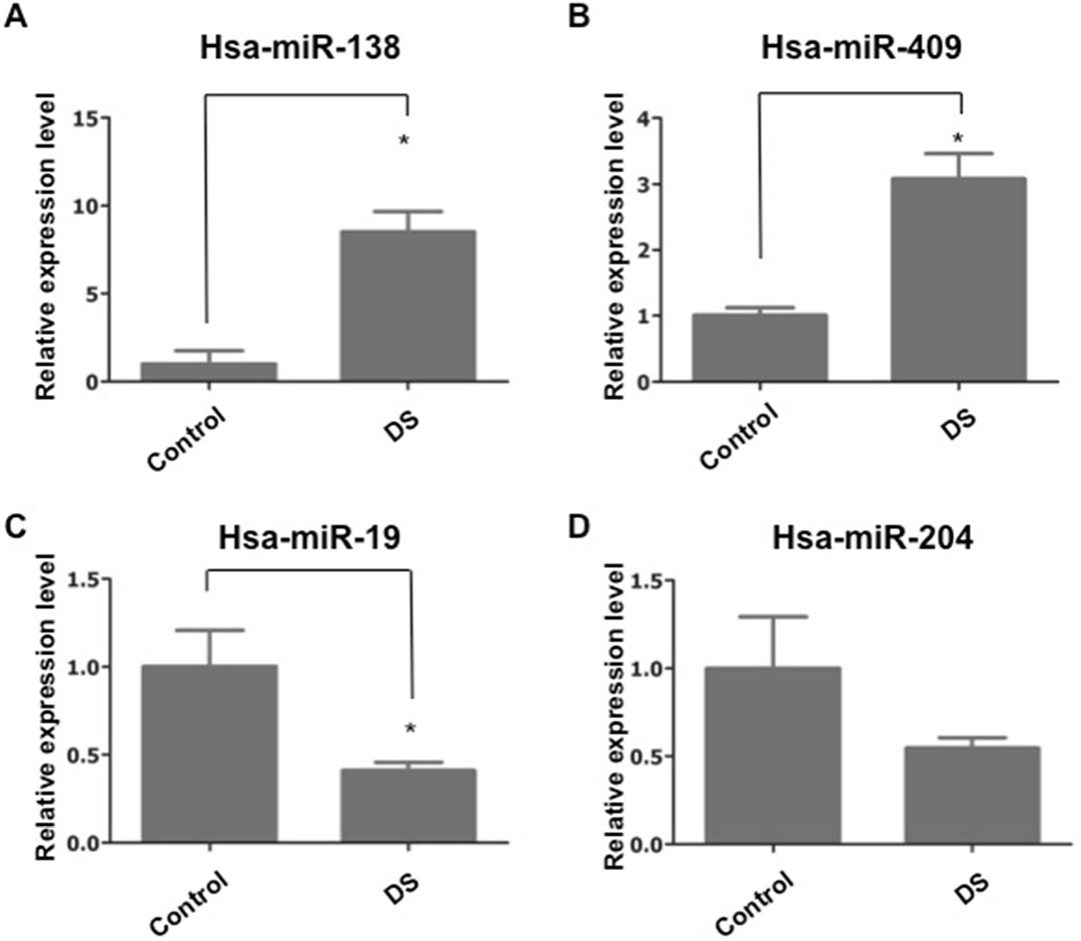
miRNA expression level was examined by real-time PCR. The expression of hsa-miR-138 (**a**), hsa-miR-409 (**b**), hsa-miR-19 (**c**) and hsa-miR-204 (**d**) were tested in fetal hippocampus tissues of DS and control group. The expression of hsa-miR-138 and hsa-miR-409 were obviously increased in DS group compared to control group. The expression of hsa-miR-19 and hsa-miR-204 were reduced in DS group compared to control group. The expression of hsa-miR-138, hsa-miR-409 and hsa-miR-19 showed significant difference between two groups. The miRNAs expression was normalized to U6 expression. The asterisks indicate the statistically significant difference

**Fig. 6:**
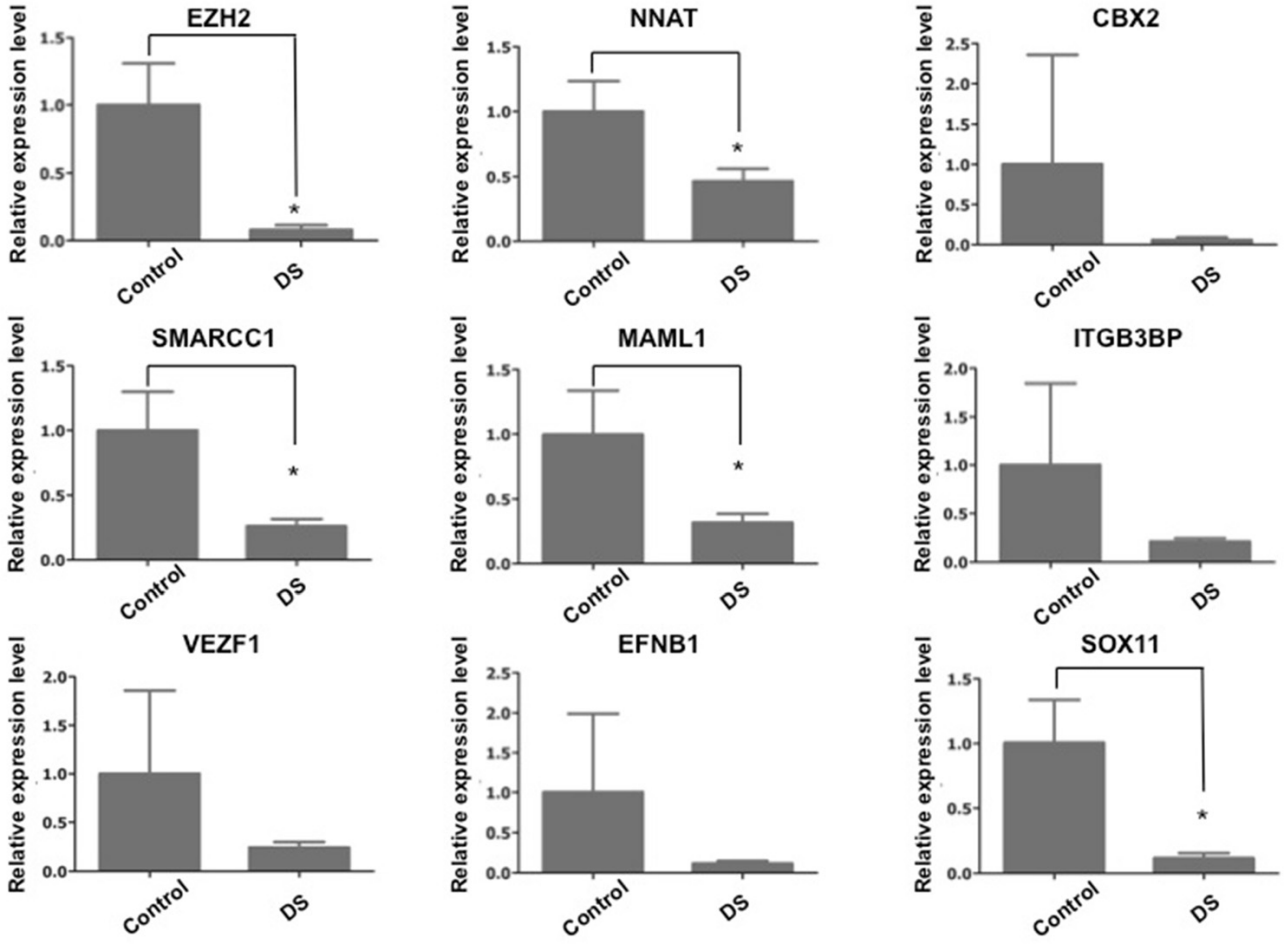
The predicted targets of hsa-miR-138 and hsa-miR-409 were reduced examined by real-time PCR. The selected mRNA level was tested in fetal hippocampus tissues of DS and control group. All the selected mRNA was reduced in DS group compared to control group. The mRNAs expression was normalized to GAPDH. The expression of *EZH2*, *NNAT, SMARCC1, MAML1* and *SOX11* showed significant difference between two groups. The asterisks indicate the statistically significant difference

**Fig. 7:**
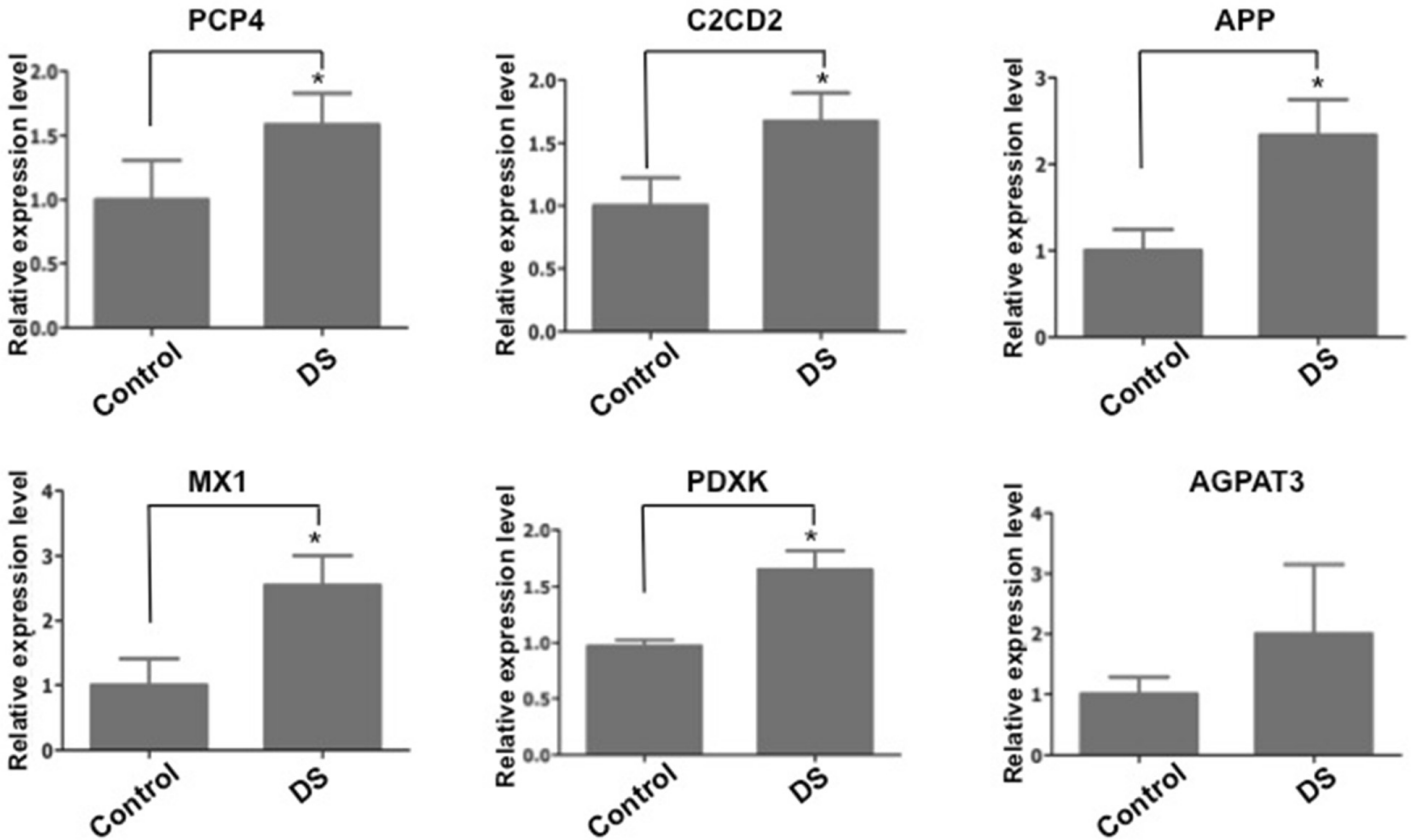
mRNA expression level was examined by real-time PCR. The selected mRNA located on chromosome 21, including *MX1, APP, AGPAT3 and PDXK*, were increased in DS group compared to control group. The mRNAs expression was normalized to GAPDH. *PCP4* and *C2CD2* were also increased in DS group. The expression of *MX1, APP, PDXK*, *PCP4* and *C2CD2* showed significant difference between two groups. The asterisks indicate the statistically significant difference

Based on the correlation analysis of miRNA and mRNA expression, the significance levels of miRNA and mRNA microarray data and previous literature, four miRNAs and 15 mRNAs, including *MAML1*, *NNAT*, *VEZF1*, *ITGB3BP*, *SOX11*, *EZH2*, *EFNB1*, *CBX2*, *SMARCC1*, *PCP4*, *C2CD2*, *APP*, *MX1*, *PDXK* and *AGPAT3* were chosen to confirm their altered expression in DS fetal compared to controls by real-time PCR. Consistent with the microarray data, real-time PCR confirmed that hsa-miR-138 and hsa-miR-409-5p were significantly increased, hsa-miR-19-3p and hsa-miR-204-5p were decreased in DS fetal hippocampal tissues compared with controls. Relative expression levels of the selected miRNAs are depicted in Fig. 5. In line with our microarray data, expression of *MAML1*, *NNAT*, *VEZF1*, *ITGB3BP*, *SOX11*, *EZH2*, *EFNB1*, *CBX2, SMARCC1* was significantly decreased in DS fetal compared to controls (Fig. 6). The expression of *MX1, APP, AGPAT3 and PDXK* that located on chromosome 21 were increased in DS group vs. control group (Fig. 7), thereby confirming results of previous studies published elsewhere [17]. Consistent with the microarray data, *PCP4* and *C2CD2*, were also increased in DS group (Fig. 7).

### EZH2 is a direct target of hsa-miR-138

**Fig. 8:**
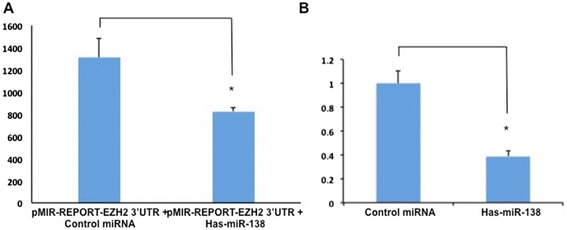
Hsa-miR-138 downregulated EZH2 by interacting with its 3′UTR. **a** The pMIR-REPORT-EZH2 3′ UTR was cotransfected into HEK293T cells with hsa-miR-138 mimics or none-target control. **b** the expression of EZH2 mRNA was tested by real-time PCR. GAPDH was served as an internal control

EZH2 had a pupative hsa-miR-138 binding site with its 3′UTR. To test whether EZH2 was a direct target of hsa-miR-138, a construct of 3′UTR of EZH2 fused downstream of the firefly luciferase gene was generated. pMIR-REPORT-EZH2 3′ UTR was transfected with hsa-miR-138 mimics or none-target control together into HEK293T cells. The results showed that hsa-miR-138 overexpression reduced the EZH2 3′UTR luciferase reporter activity compared to none-target control transfected group (Fig. 8a). In addition, real-time PCR analysis showed that hsa-miR-138 overexpression significantly decreased EZH2 mRNA level in HEK293T cells (Fig. 8b). These results indicated that EZH2 was a direct target of hsa-miR-138 in HEK293T cells.

## Discussion

Down syndrome is one of the most common chromosomal abnormalities among live born infants. The major neurological deficits that afflict DS individuals are intellectual disability and the early onset of Alzheimer’s disease [4]. Numerous abnormalities in the DS nervous system have been reported. In nervous system, the onset of Alzheimer’s disease has been intensively studied, while the mechanisms of intellectual disability in DS patients have not been well elucidated. The goal of our study was to establish a comprehensive picture of miRNA-mRNA network that could cause neurological deficiency especially intellectual disability in fetal Down syndrome. To our best knowledge, this study is the first whole-genome miRNA and mRNA expression microarray profiling in hippocampal tissues of human fetal Down syndrome, will therefore provide a clue to study the mechanisms of neurological deficiency occurred in DS patients.

MicroRNAs are known to regulate the expression of genes post-transcriptionally. They are involved in a wide range of biological processes, such as proliferation, apoptosis, tumor development, stress response etc. In our study, the differentially expressed miRNAs in hippocampus of DS fetal suggested an association with DS neurological problems. Based on the amplified developmental instability hypothesis [18] of DS that a number of trisomic genes result in a genetic imbalance, we assumed that these differentially expressed miRNAs participated in the complicated genetic imbalance in DS patients. Correlation analysis between miRNA and the predicted target mRNA expression levels showed that hsa-miR-138 were involved most frequently in significantly inversely correlated miRNA-target mRNA pairs. The hsa-miR-138 has been previously demonstrated to have essential roles in tumor suppression. It was down-regulated in various cancers [19, 20]. In this research, we found that hsa-miR-138 is up-regulated in hippocampus from DS fetal. The function of hsa-miR-138 in cell proliferation and cell apoptosis may affect the normal development of hippocampus. In our study, the expression of hsa-miR-155 and let-7c that from chromosome 21 also were increased in DS group. But there was no significant difference between two groups, which is probably due to the small sample size and sample variability.

The EZH2 protein is the catalytic core protein in the Polycomb group (PcG) repressive complex 2 (PRC2), which are involved in epigenetic regulation of gene expression. EZH2 plays important roles in the progress of cell fate decision, cell cycle regulation, senescence, cell differentiation and cancer. EZH2, has highly conserved putative hsa-miR-138 binding site within its 3′UTR, has been proved as one of directly targets of hsa-miR-138 [21]. The functional role of EZH2 has already been studied extensively. In animal model, it was shown that EZH2, which formed a PcG complex with EED (embryonic ectoderm development), could induce an ectopic neural axis in neural induction [22]. The mutation of EZH2 were related to primary central nervous system lymphoma [23]. Moreover, muted EZH2 led to Weaver Syndrome that had intellectual disability as one of its common features [24]. In this research, EZH2 is downregulated in hippocampal tissues in DS versus control samples, which implied that EZH2 also play roles in neurological deficiency of DS especially in intellectual disability.

Amyloid beta/A4 precursor protein (APP) has been shown essential roles in Alzheimer’s disease (AD) [25, 26] and in autism [27]. In this research, APP was up-regulated that implied its potential role in Down Syndrome.

In this research, we focused on the miRNA and its predicted mRNA in hippocampus of DS. There are some limitations to our study. Firstly, we used a relatively small sample size. Microarray analysis was performed using the RNA extracted from three samples in each group, and the validation experiments were performed using four samples in DS group and three samples in control group. However, significant differences could be identified in gene expression. Secondly, correlation analysis suggested a highly significant negative correlation between expression levels of hsa-miR-138 and its potential target mRNAs of interest. We need to further determine whether these mRNAs are targeted by endogenous miRNAs in hippocampus. Nevertheless, our data provide a clue regarding hsa-miR-138 and its targets EZH2 are associated with neurological deficiency in DS. Further analysis of our microarray data will help us to understand the mechanism of neurological deficiency in DS patients.

## Conclusion

This research represents an integrated analysis of miRNA and mRNA expression in hippocampus of DS fetal. We identified a number of differentially expressed miRNA and its predicted mRNA, including hsa-miR-138 and its target EZH2. It is presumed that hsa-miR-138 and EZH2 could be involved in neurological deficiency of DS patients. It will help better understand intellectual disability development in DS. While the mechanisms as to how hsa-miR-138 targets EZH to play roles in DS needs to be further studied.

## Abbreviations

DS, down syndrome; EED, embryonic ectoderm development; EZH2, zeste homolog 2; MiRNAs, miRNAs; PcG, Polycomb group; PRC2, Polycomb group repressive complex 2

## Additional files

Additional file 1: Table S1. List of differentially expressed miRNA in DS group versus control group by microarray assay. Red indicates increased expression of miRNA and green indicates decreased expression of miRNA in DS group versus control group. (XLSX 102 kb) Additional file 2: Figure S1. Heatmap of mRNA expression in fetal hippocampus of DS fetal cohorts and control cohorts. Hierarchical clustering of all samples based on the log2 expression values of all differentially expressed mRNAs. Samples are shown in the columns and mRNAs in the rows. The boxes in color indicate the log2 intensities of the mRNAs, with green indicating low expression and red indicating high expression. (JPG 455 kb)
