# Supplementary figures and images for: Integrated miRNA and mRNA expression profiling in fetal hippocampus with Down syndrome
Source: J Biomed Sci. 2016 Jun 7;23:48. doi: 10.1186/s12929-016-0265-0 (PMC4897952; doi:10.1186/s12929-016-0265-0)

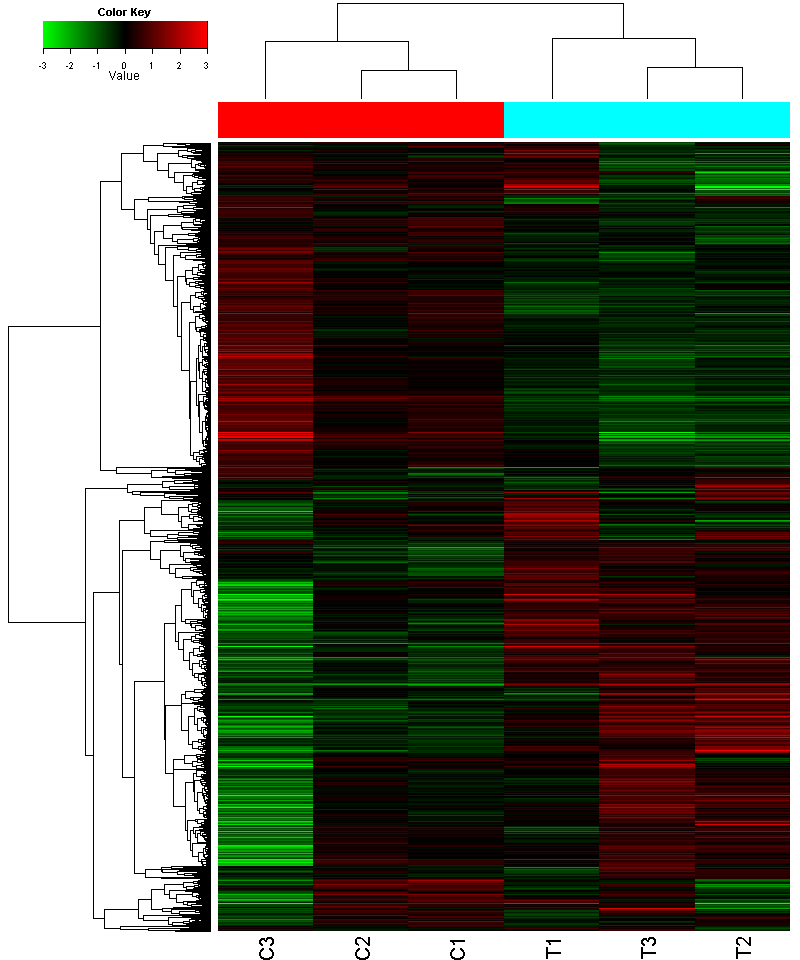

Supplement: Additional file 2: Figure S1. — Heatmap of mRNA expression in fetal hippocampus of DS fetal cohorts and control cohorts. Hierarchical clustering of all samples based on the log2 expression values of all differentially expressed mRNAs. Samples are shown in the columns and mRNAs in the rows. The boxes in color indicate the log2 intensities of the mRNAs, with green indicating low expression and red indicating high expression. (JPG 455 kb) [file 12929_2016_265_MOESM2_ESM.jpg]
